# LRBA Deficiency Can Lead to Lethal Colitis That Is Diminished by SHIP1 Agonism

**DOI:** 10.3389/fimmu.2022.830961

**Published:** 2022-05-04

**Authors:** Raki Sudan, Sandra Fernandes, Neetu Srivastava, Chiara Pedicone, Shea T. Meyer, John D. Chisholm, Robert W. Engelman, William G. Kerr

**Affiliations:** ^1^ Department of Microbiology and Immunology, SUNY Upstate Medical University, Syracuse, NY, United States; ^2^ Department of Chemistry, Syracuse University, Syracuse, NY, United States; ^3^ Department of Pathology and Cell Biology, University of South Florida, Tampa, FL, United States; ^4^ Department of Pediatrics, University of South Florida, Tampa, FL, United States; ^5^ H. Lee Moffitt Comprehensive Cancer Center & Research Institute, University of South Florida, Tampa, FL, United States

**Keywords:** LRBA deficiency, SHIP1, src homology 2 domain-containing inositol phosphatase 1, colitis, ILC3 cytokines

## Abstract

Humans homozygous for inactivating *LRBA* (lipopolysaccharide (LPS)-responsive beige-like anchor) mutations or with compound heterozygous mutations exhibit a spectrum of immune-related pathologies including inflammatory bowel disease (IBD). The cause of this pathology remains undefined. Here we show that disruption of the colon epithelial barrier in LRBA-deficient mice by dextran sulfate sodium (DSS) consumption leads to severe and uniformly lethal colitis. Analysis of bone marrow (BM) chimeras showed that susceptibility to lethal colitis is primarily due to LRBA deficiency in the immune compartment and not the gut epithelium. Further dissection of the immune defect in LRBA-deficient hosts showed that LRBA is essential for the expression of CTLA4 by Treg cells and IL22 and IL17 expression by ILC3 cells in the large intestine when the gut epithelium is compromised by DSS. We further show that SHIP1 agonism partially abrogates the severity and lethality of DSS-mediated colitis. Our findings indicate that enteropathy induced by LRBA deficiency has multiple causes and that SHIP1 agonism can partially abrogate the inflammatory milieu in the gut of LRBA-deficient hosts.

## Introduction

*Lrba* gene was initially identified by gene trapping of a lipopolysaccharide (LPS)-inducible gene in 70Z/3 pre-B cells (1) and subsequently shown to be a CHS/beige-like gene whose expression is induced in both B lymphocytes and macrophages by LPS (2). Comprehensive homology analysis of orthologs from multiple species led to a molecular model for LRBA function that proposes that it facilitates vesicle trafficking of immune effector molecules in coordination with signaling at plasma membrane-localized receptors (2). This model has been confirmed and extended in several different species including *Caenorhabditis elegans* (3). The recent development and analysis of LRBA-deficient mice have also supported this function for LRBA, as γ-IFN production and Granzyme B secretion are compromised in LRBA-deficient NK cells when activating receptors engage ligands on a target cell (4). LRBA is also expressed by epithelial tissues where its expression increases following the acquisition of the malignant state. In epithelial cancers, LRBA acts downstream of EGF-R to promote cell survival (5). LRBA may be essential for human breast cancer cell survival, as its increased expression is a prominent component of a gene signature that predicts poor prognosis and response to therapy in breast cancer patients (6, 7).

The recent identification of human kindreds possessing inactivating *LRBA* mutations indicates an essential role in immune regulation, as these patients exhibit a varied spectrum of both autoimmune pathologies and immune deficiencies such as defective antibody production and humoral responses (8, 9). However, hypogammaglobulinemia is not uniformly observed in all LRBA-deficient patients (10), while autoimmunity, and specifically inflammatory bowel disease (IBD), is observed in a significant proportion of these patients (8–12).

Further confirming a role in susceptibility to colitis in mammals, it was recently shown that an LRBA-deficient mouse variant, created by ENU mutagenesis, exhibits increased sensitivity to dextran sulfate sodium (DSS)-mediated colitis at doses of DSS that are not lethal to the host (13). The authors also found that LRBA-deficient dendritic cells (DC) exhibited increased PI3K/mTORC1 signaling and were hyper-responsive to PAMP ligands, namely, ligands for the endosomal toll-like receptors (TLRs) 3, 7, and 9, suggesting that gut DC may create a hyper-inflammatory environment that culminates in colitis after DSS challenge (13). The increased PI3K/Akt signaling observed by Wang et al. in their LRBA-deficient *oscar* strains (13) indicates a potential role for regulation by SHIP1—an important regulator of PI3K-Akt signaling (14) that is known to regulate many aspects of macrophage and DC signaling (15–17). Thus, activating SHIP1 signaling might have a protective role in LRBA deficiency.

It was shown that LRBA regulates CTLA4 levels by preventing its lysosomal degradation and allowing its recycling to the plasma membrane of activated T cells and Treg cells. In LRBA-deficient patients, the activated T cells and Treg cells have reduced levels of immunoregulatory molecule CTLA4 due to increased lysosomal degradation, suggesting that autoimmune pathologies might also result from defective immunoregulation (18, 19). Chloroquine treatment, which inhibits lysosomal degradation, rescued surface expression of CTLA4 in LRBA-deficient patients’ T cells (18). Whether this molecular defect contributes to enhanced susceptibility to IBD in LRBA patients is not clear; however, given the prominent role that Treg cells play in maintaining gut homeostasis, this seems a likely scenario (20). However, Wang et al. did not observe decreased CTLA4 expression by intestinal Treg cells in their ENU-induced model of LRBA deficiency, suggesting that there could be dysfunction of other immune cells at play in IBD caused by LRBA deficiency (13).

Consistent with the proposed role for LRBA in immune regulation, the treatment of LRBA-deficient patients with abatacept, a CTLA4-Ig fusion drug, enabled significant clinical improvement (18). Sirolimus (rapamycin) has also been used to control autoimmune disease in LRBA-deficient patients (21). However, allogeneic bone marrow (BM) transplantation (BMT) has also been used to treat LRBA deficiency with long-term remission being reported post-transplant, particularly in patients who were transplanted prior to significant disease emerging (22). The success of allogeneic BMT as a treatment for LRBA deficiency may be due in part to the profound defect in the ability of the LRBA host to reject an allogeneic BM graft and their relative resistance to graft-versus-host disease (GvHD) (4).

Using a severe form of DSS-induced colitis that is lethal in a minority of wild-type (WT) hosts, we show that LRBA knockout (LRBA KO) mice are uniformly susceptible to DSS-mediated lethal disease. We show that susceptibility to DSS colitis is not due to epithelial loss of LRBA expression or alterations in the microbiota. We confirm that LRBA KO mice have significantly reduced expression of CTLA4 in Treg cells but also that reduced IL17 and IL22 production by ILC3 in the gut may further exacerbate DSS-induced colitis. Finally, we show that SHIP1 agonism can extend the survival of LRBA-deficient hosts following the DSS challenge.

## Materials and Methods

### Mice

LRBA KO mice (129Sv) were described previously (4). 129Sv WT mice were purchased from Taconic Bioscience and were bred and maintained at an institutional animal facility. A DSS challenge study was also performed in LRBA KO progeny and their WT littermates on a C57BL6 background obtained after backcrossing LRBA KO (129Sv) mice to C57BL/6NTac mice for 7 generations.

### Induction of Experimental Colitis

LRBA KO and WT mice were given 3% (w/v) DSS (MW 36,000–50,000, MP Biomedicals, Santa Ana, CA, USA) in drinking water for 7 days. Mice received fresh DSS every other day, and after 7 days, DSS water was replaced with normal water. Mice were monitored for weight loss, rectal bleeding, and survival. Mice that lost more than 30% of their initial body weight or became moribund were euthanized. Stool score or rectal bleeding was assessed as described (23). For the study of SHIP1 agonism in DSS-induced lethality, mice were treated with AQX-MN100 (3 mg/kg) or vehicle (5%ETOH:H20) daily by oral gavage (10 µl/g) beginning 1 day prior to consumption of DSS-containing drinking water and then for 7 additional days.

### Histological Analysis

Colonic tissues were assigned histologic grades of colitis based on the degree of epithelial crypt damage, the presence of edema, goblet cell depletion, and/or inflammatory cell infiltration. Epithelial crypt damage and polymorphonuclear leukocyte (PMN) infiltration were graded individually in each colonic tissue and summed using scoring criteria established previously in SHIP1^−/−^ mice (24).

### Bone Marrow Chimera Experiments

Lethally irradiated WT or LRBA^−/−^ mice were injected *via* i.v. injection with 3–4 × 10^6^ LRBA KO or WT BM cells as indicated. Eight weeks after the transplant, mice were given 3% DSS in drinking water for 7 days, and disease was assessed.

### AQX-MN100 Synthesis

AQX-MN100 was prepared following the previously reported route with minor modifications (25). The purity and identity of AQX-MN100 were confirmed by a combination of mass spectrometry, NMR, and combustion analysis. Further details concerning the synthetic route and confirmation of purity and identity can be provided upon request.

### Cohousing

Age- and gender-matched LRBA KO and WT mice were cohoused for 2 months at 2:3 or 3:2 ratios and were then given 3% DSS in drinking water for 7 days, and their disease was evaluated as above.

### Cell Isolation and Flow Cytometry

Large intestine (LI) lamina propria cells were isolated by following the protocol described previously (26). For Treg analysis and CTLA4 expression on Treg cells, single-cell suspensions were stained with CD3 (145-2C11, eBioscience, San Diego, CA, USA), CD4 (RM4-5 (BD Biosciences, San Jose, CA, USA), and CD25 (PC61.5, eBioscience) antibodies followed by intracellular staining with FoxP3 (FJK-16s, eBioscience) and CTLA4 (UC10-4B9, BioLegend, San Diego, CA, USA) antibodies using FoxP3 intracellular staining buffer set (eBioscience). For ILC3 analysis, LI lamina propria cells were incubated for 4 h in complete Roswell Park Memorial Institute (RPMI) media in the presence of phorbol myristate acetate (PMA) (200 ng/ml), ionomycin (1 μg/ml), and BD GolgiStop and GolgiPlug. Cells were surface stained with mouse lineage antibody cocktail (BD), CD45 (30-F11, BD), Thy-1.2 (53-2.1, eBioscience), IL7Rα (A7R34 (BioLegend), NKp46 (29A1.4, BD), and CCR6 (29-2L17, BioLegend) antibodies followed by intracellular staining with RORγt (Q31-378, BD), IL22 (IL22JOP, eBioscience), and IL17a (eBio17B7, eBioscience). Flow cytometry was performed using BD LSR FORTESSA, and data analysis was performed using FlowJo software.

### Statistical Analyses

All statistical analyses were performed using Graph Pad Prism. Unpaired two-tailed Student’s t-tests were used to analyze the significance of data sets. Survival was analyzed using the Mantel–Cox log-rank test or a Kaplan–Meier log-rank test. A p-value <0.05 was considered statistically significant.

## Results

### LRBA-Deficient Mice Are Highly Susceptible to Dextran Sulfate Sodium Colitis

**Figure 1:**
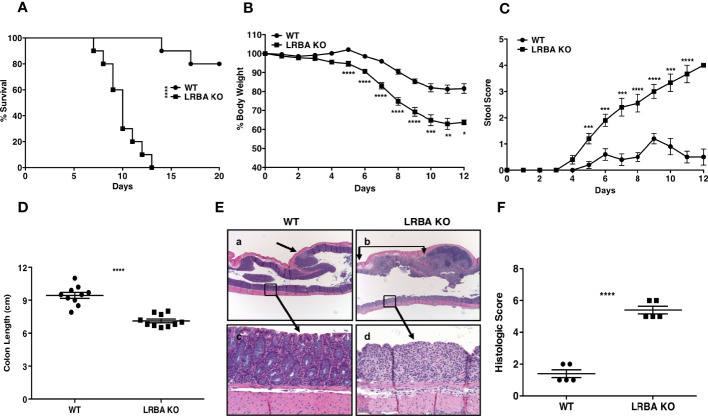
LRBA KO mice are highly susceptible to DSS colitis. WT and LRBA KO mice (129Sv background) were given 3% DSS in drinking water for 7 days. **(A)** Survival plot, **(B)** % weight loss, and **(C)** Stool score based upon rectal bleeding of WT and LRBA KO mice. Mice were euthanized by the end of 7 days for colon length measurement and histologic analysis. **(D)** Colon length, **(E)** representative histologic appearance of colitis in WT mice (a, c) with grade 2 colitis comprised of focal, mild epithelial crypt damage and focal <25 PMN/hpf infiltration of the lamina propria (a, arrow) but with much of the colonic mucosa without epithelial damage, goblet cell depletion, edema, or PMN infiltration (c), as compared to LRBA KO mice (b, d) with grade 5 colitis comprised of regional, marked epithelial erosion, crypt loss, goblet cell depletion (d), submucosal edema (b, between arrows), and moderate to marked >25 PMN/hpf infiltration of the lamina propria and submucosa. **(F)** Histologic scoring of colitis in WT and LRBA KO mice. **(A–D)** Pooled data from 2 independent experiments with total n = 10. **(E, F)** Data from 1 of 2 independent experiments are shown with n = 5. *p < 0.05, **p < 0.01, ***p < 0.001, ****p < 0.0001. KO, knockout; DSS, dextran sulfate sodium; WT, wild type; PMN, polymorphonuclear leukocyte.

Under homeostatic conditions, we did not observe any major histological abnormality in any of the major organs tested in LRBA-deficient mice including SI (4). Also, we performed histopathology analysis of SI and LI on a cohort of aged LRBA-deficient mice and did not observe any pathology. We previously found that LRBA KO mice have a small, but significant, increase in Treg numbers in the small intestine (4), but not in the spleen. This suggested inflammatory pressure in the intestine during normal physiology in LRBA KO mice. To assess this possibility, we further stressed the immunoregulatory capacity in the gut immune compartment by compromising the intestinal epithelial barrier *via* administration of 3% DSS (a dose of DSS that is lethal to some WT hosts) to both LRBA KO and WT hosts (**Figure 1**). *Ad libitum* administration of 3% DSS in drinking water to LRBA-deficient hosts for 7 days was 100% lethal after 12 days, while only ~20% of WT hosts succumbed. This suggested a stark difference in the capacity of LRBA KO hosts to manage inflammatory stress in the intestine (**Figure 1A**). This was confirmed by further analysis of clinical parameters, as 3% DSS dosing also resulted in significantly greater weight loss during the study (**Figure 1B**) and rectal bleeding (**Figure 1C**) based on a stool scoring criteria (23). The pathological analysis confirmed the dramatic impact of DSS challenge in LRBA KO hosts, as they had significant compaction of their colons (**Figure 1D**) and dramatic destruction and leukocytic infiltration in the LI as compared to WT mice (**Figures 1E, F** **)**. We observed no evidence of inflammation in the LI of LRBA KO mice that were not exposed to DSS-containing drinking water (**Supplementary Figure 1**). These findings demonstrate that severe, life-threatening autoimmune disease can occur in LRBA KO mice and indicate a crucial role for LRBA in the maintenance of homeostasis in the gut.

### LRBA Deficiency in the Hematopoietic Compartment Renders Mice Susceptible to Colitis

**Figure 2:**
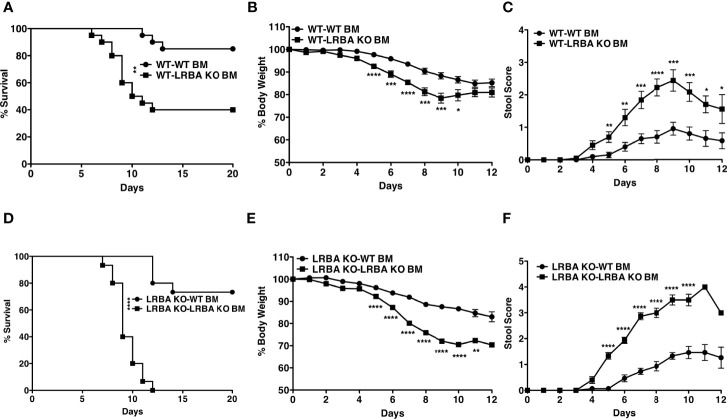
LRBA deficiency in the hematopoietic compartment renders mice susceptible to colitis. Lethally irradiated WT mice (129Sv) were reconstituted with WT or LRBA KO BM (129Sv). After 8 weeks, mice were given 3% DSS for 7 days. **(A)** Survival plot of WT mice reconstituted with WT BM cells (WT-WT BM) vs. WT mice reconstituted with LRBA KO BM cells (WT-LRBA KO BM), **(B)** % weight loss, and **(C)** stool score. Lethally irradiated LRBA KO mice were reconstituted with WT or LRBA KO BM. After 8 weeks, mice were given 3% DSS for 7 days. **(D)** Survival plot, **(E)** % weight loss, and **(F)** stool score of LRBA KO mice reconstituted with WT BM cells (LRBA KO-WT BM) or LRBA KO BM cells (LRBA KO-LRBA KO BM). **(A–E)** Pooled data from 2 independent experiments. For panels **(A–C)**, n = 20, whereas n = 15 for **(D–F)**. *p < 0.05, **p < 0.01, ***p < 0.001, ****p < 0.0001. WT, wild type; KO, knockout; DSS, dextran sulfate sodium; BM, bone marrow.

In order to determine if LRBA deficiency in the gut epithelium or the hematolymphoid system causes increased susceptibility to intestinal inflammatory disease, we constructed BM chimeras where lethally irradiated WT hosts were reconstituted with BM from LRBA KO or WT donors. After allowing 8 weeks for BM reconstitution of the WT host’s immune system, we then performed a DSS challenge. We found that WT hosts reconstituted with LRBA KO BM were significantly more sensitive to DSS-mediated lethal disease (**Figure 2A**) and exhibited more severe disease as exhibited by increased body weight loss (**Figure 2B**) and stool scoring (**Figure 2C**). We then performed the reciprocal study where lethally irradiated LRBA KO hosts were reconstituted with BM from WT or LRBA KO donors. As with germline LRBA KO hosts, LRBA KO hosts reconstituted from LRBA KO BM were uniformly susceptible to lethal disease with all such chimeras succumbing by 12 days (**Figure 2D**). Importantly, LRBA KO hosts reconstituted from WT BM donors were resistant to DSS-mediated lethal disease and showed comparable survival to WT hosts (**Figure 2D**). The LRBA KO hosts reconstituted with WT BM also showed significantly improved clinical responses as they lost less body weight (**Figure 2E**) and showed decreased evidence of rectal bleeding based on stool scoring (**Figure 2F**). These results demonstrate that LRBA deficiency in the hematolymphoid system is necessary for susceptibility to disease and implied that there is an immune defect that causes susceptibility to colitis and not a defect in gut epithelial function. Also, when we combined data for survival from all four conditions and compared the survival of WT mice reconstituted with WT BM (non-hematopoietic compartment of WT origin) vs. LRBA KO mice reconstituted with WT BM (non-hematopoietic compartment of LRBA KO origin) and both these conditions have a hematopoietic compartment of WT origin), we did not observe any statistical significance, thus suggesting that LRBA-deficient non-hematopoietic compartment alone is not sufficient for disease susceptibility (**Figures 2A, D** and **Supplementary Figure 2**). However, when we compare LRBA KO hosts reconstituted with LRBA KO BM vs. WT hosts reconstituted with LRBA KO BM, we did observe that LRBA KO mice reconstituted with LRBA KO BM are more susceptible compared to WT mice reconstituted with LRBA KO BM. This suggests that LRBA deficiency in the non-hematopoietic compartment can enhance disease susceptibility when you lack LRBA in the hematopoietic compartment indicating that there could be some synergistic effect between the hematopoietic compartment and non-hematopoietic compartment under LRBA-deficient condition (**Supplementary Figure 2**).

### Susceptibility to Colitis Observed in LRBA-Deficient Mice Is Non-Transmissible

**Figure 3:**
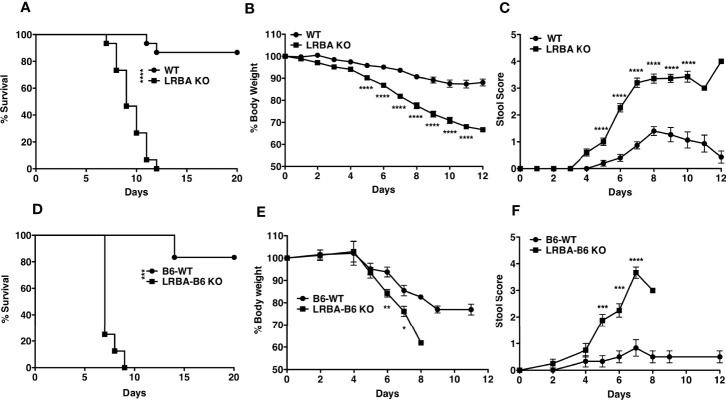
Susceptibility to colitis observed in LRBA KO mice is non-transmissible. WT and LRBA KO (129Sv background) mice co-housed for 2 months received 3% DSS for 7 days. **(A)** Survival, **(B)** % weight loss, and **(C)** stool score. Pooled data from 2 independent experiments n = 15. WT and LRBA KO littermates on a C57BL6 background (F7 backcross) received 3% DSS for 7 days. **(D)** Survival, **(E)** weight loss, and **(F)** stool score. *p < 0.05, **p < 0.01, ***p < 0.001, ****p < 0.0001. KO, knockout; DSS, dextran sulfate sodium; WT, wild type.

The microbiota present in the intestine can have a profound influence on gut homeostasis and inflammation in the gut (27). It was conceivable that LRBA deficiency may have rendered such hosts susceptible to colonization by bacterial species that then contribute to a severe and lethal reaction following DSS-induced damage to the mucosal epithelial barrier. To test this possibility, we co-housed WT and LRBA KO hosts for 2 months. Co-housing for this extended period enables microorganisms that might be present in hosts of one genotype to colonize hosts of the other genotype. Following co-housing, we then performed the same DSS challenge. We found that co-housing did not reverse the acute sensitivity of LRBA KO hosts to DSS, as they died rapidly and uniformly with the same kinetics as we observed without co-housing (**Figure 3A**). Importantly, WT hosts co-housed with LRBA KO hosts maintained their relative resistance to DSS (**Figure 3A**). Consistent with LRBA KO hosts having compromised survival after the DSS challenge, the co-housed LRBA KO hosts also lost significantly more body weight (**Figure 3B**) and had greater stool scores (**Figure 3C**) relative to co-housed WT hosts. In the above co-housing study, mice of each genotype were co-housed after reaching adulthood; however, it is now believed that the microbiome is established postpartum. As the microbiota becomes established soon after birth, we sought to test whether the LRBA mutation might render WT hosts sensitive when they are co-housed with LRBA KO littermates since birth, as they will have been exposed to each other’s microbiomes postpartum and thus have essentially the same microbiome. To do this comparison, we took advantage of a recent variant of our LRBA KO mice that has been backcrossed seven times to a C57BL6 background (F7). These F7 LRBA KO mice were generated from matings of LRBA^+/−^ partners on a C57BL6 background and thus have been co-housed since birth with WT littermates and in some instances shared the same mother. As seen with LRBA KO (129v) hosts, the DSS challenge was uniformly lethal in LRBA KO (BL6) hosts, but not their WT littermates (**Figure 3D**), and this was correlated with significant weight loss (**Figure 3E**) and stool score (**Figure 3F**). In fact, the onset of death in LRBA KO hosts on a C57BL6 background relative to a 129Sv background is accelerated with all BL6 LRBA KO hosts expiring by 9 days after DSS administration vs. 12 days on the 129Sv background. These findings indicate that the severe colitis susceptibility of LRBA KO hosts is not defined by their microbiome and is highly penetrant across different murine genetic backgrounds.

### LRBA Knockout Mice Show Reduced CTLA4 Expression on Treg cells and Impaired ILC3 Effector Function Following Colonic Stress

**Figure 4:**
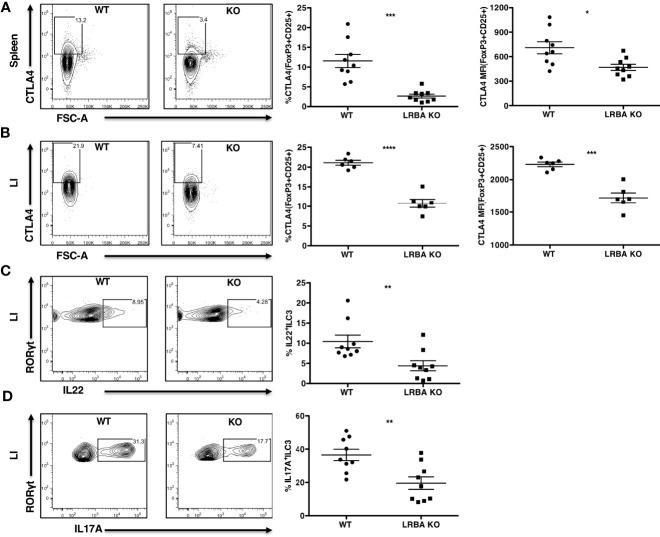
LRBA KO mice show reduced CTLA4 expression on Treg cells and impaired ILC3 effector function following colonic stress. WT and LRBA KO mice received 3% DSS in drinking water for 5 days and were analyzed for intracellular CTLA4 expression and IL22 and IL17A production by ILC3. **(A)** Contour plots and scatter plots showing CTLA4 expression on splenic FoxP3^+^CD25^+^ Treg cells after gating on viable CD3^+^CD4^+^ cells. **(B)** Plots showing CTLA4 expression on LI lamina propria FoxP3^+^CD25^+^ Treg cells gated on viable CD3^+^CD4^+^ cells. Plots showing the frequency of IL22^+^RORγt^+^ **(C)** and IL17A^+^RORγt^+^ ILC3 **(D)** after gating on viable Lin^−^CD45^+^Thy1.2^+^IL7Rα^+^RORγt^+^ cells in the LI lamina propria. Data pooled from 3 independent experiments **(A, C, D)**, n = 9 per genotype. **(B)** Pooled from 2 independent experiments, n = 6 per genotype. *p < 0.05, **p < 0.01, ***p < 0.001, ****p < 0.0001. KO, knockout; WT, wild type; DSS, dextran sulfate sodium; LI, large intestine.

Our BM transfer studies indicated that LRBA deficiency in the immune compartment is likely responsible for susceptibility to colitis and suggested that LRBA may be required for the function of an immune regulatory cell and/or an innate lymphocyte cell type capable of controlling microbial dysbiosis that results from DSS-induced epithelial permeability. CTLA4 expression by Treg cells (28–31) and IL22 and IL17 production by ILC3 innate lymphocytes (32–35) have both been shown to prevent or limit the development of inflammatory bowel syndromes and particularly colitis in the LI. Thus, we examined the presence of these cell types and their expression of CTLA4, IL22, and IL17 in LRBA KO mice during the DSS challenge as compared to WT mice (**Figure 4**). We found that Treg cell numbers are unchanged in the LI during both normal homeostases and after the DSS challenge; however, the frequency of Treg cells is slightly increased in the spleens of LRBA KO hosts vs. WT controls but interestingly only upon DSS challenge (**Supplementary Figure 3**). However, the capacity of both splenic (**Figure 4A**) and LI Treg cells (**Figure 4B**) in LRBA KO hosts after the DSS challenge to express CTLA4 was significantly reduced as compared to Treg cells in WT hosts, and this was evident in both the frequency of CTLA4^+^ Treg cells and also in the surface density of CTLA4 on Treg cells in the LI (**Figures 4A, B** and **Supplementary Figure 4**). We also observed significant reductions in both IL22 (**Figure 4C**) and IL17 (**Figure 4D**) production by RORγt+ ILC3 present in the LI of LRBA^−/−^ hosts as compared to WT hosts during the DSS challenge. LRBA deficiency does not appear to compromise the development of Lin^−^CD45^+^Thy1.2^+^IL7Rα^+^RORγt^+^ ILC3, as this cell population is present in comparable numbers in LRBA KO hosts as compared to WT hosts during the DSS challenge (**Supplementary Figure 5**). Thus, rather than limiting the development, trafficking, or formation of key immunoregulatory cell types in the gut, LRBA has a precise role in promoting the surface deposition and secretion of key immunoregulatory and effector molecules (e.g., IL17 and IL22), as we also observed previously for γ-IFN production by NK cells (4).

### SHIP1 Agonism Prolongs Survival of LRBA-Deficient Hosts in Dextran Sulfate Sodium-Induced Colitis

**Figure 5:**
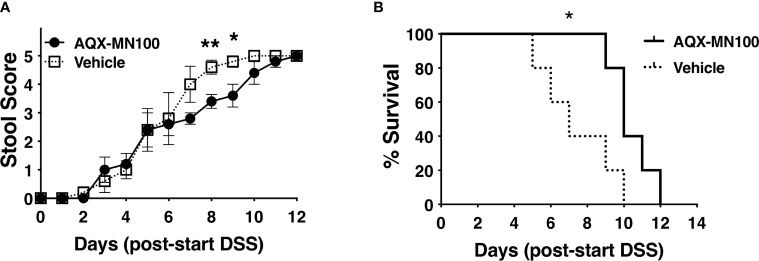
SHIP1 agonism extends survival of LRBA-deficient mice in a lethal DSS-colitis model. Mice were placed on DSS-containing drinking water for 7 days and dosed with AQX-MN100 (3 mg/kg) or vehicle (n = 5/group) *via* daily oral gavage (10 ml/g) for 8 days beginning 1 day prior to exposure to DSS-containing drinking water. **(A)** Stool score was monitored daily and ranked at 5 when a mouse was euthanized based on rectal bleeding and weight loss (two-tailed Student’s t-test was used to compare mice on each day, **p < 0.01, *p < 0.05). **(B)** Survival of the two cohorts was compared by the Kaplan–Meier log-rank test (*p < 0.05). DSS, dextran sulfate sodium.

A recent study implicated myeloid-derived DC as a cell type required to trigger DSS-mediated colitis in the gut of the *oscar* strains of LRBA deficiency identified as part of an ENU mutagenesis screen for colitis susceptibility loci (13). Wang et al. proposed colitis in the *oscar* strain results from hyper-activation of the PI3K/Akt pathway in myeloid DC that SHIP1 opposes, leading to overproduction of type I IFN induced in response to TLR ligands (13). SHIP1 is known to limit signaling by all three TLRs implicated in DSS-induced colitis, including TLR-3, TLR-7, and TLR-9 (36–38). Thus, agonism of SHIP1 might potentially abrogate, or diminish, this inflammatory response *in vivo*. We then examined the possibility that SHIP1 agonism might reduce the lethality of DSS colitis by orally dosing LRBA KO mice with the SHIP1 agonist, AQX-MN100 (39), immediately prior to and during exposure to DSS-containing drinking water. We found that AQX-MN100 significantly extended survival in our lethal DSS-induced colitis model of LRBA-deficient hosts (**Figures 5A, B** **)**. These findings are consistent with those of Wang et al., who showed that hyper-activation of the PI3K/Akt pathway contributes to colitis in LRBA-deficient mice (13) but also suggest that other factors also contribute.

## Discussion

Clinical and genetic characterization of kindreds containing individuals who have homozygous or compound heterozygous inactivating *LRBA* mutations has revealed a crucial role for this gene in preventing autoimmunity in humans. There is variation in the immune defects seen in these patients, with autoimmune enteropathy being one of the more frequently found pathologies (41% or 62%, depending on the cohort) (11, 12). Initial characterization of independent murine strains homozygous for inactivating *Lrba* mutations under normal physiological conditions did not reveal the presence of detectable autoimmune disease in these mutants, despite studies confirming that murine LRBA impacted Treg expression of CTLA4 (4, 40, 41). Wang et al. using the ENU-generated *oscar* mutants in the murine *Lrba* locus observed that a non-lethal form of colitis could occur in their model with the administration of low doses of DSS in drinking water (13). In addition, a major defect in NK cell function was identified with particular relevance to allogeneic BM transplantation, suggesting that there could be innate immune defects as well, particularly with respect to the secretion of key immune effector molecules by NK cells (4). Here we confirm that LRBA’s role in preventing autoimmune disease and specifically enteropathy in the gut mucosa, the one pathology that is thus far routinely observed in LRBA-deficient patients and now in LRBA-deficient rodents. Importantly, our study increases the understanding of how LRBA is essential to the prevention of inflammatory disease in the gut. Our analysis of BM chimeras shows that this defect arises primarily from LRBA deficiency in the immune system and not due to loss of LRBA function in the gut epithelial barrier. However, in every case, we saw maximum disease susceptibility when LRBA was absent in both hematopoietic and non-hematopoietic compartments, suggesting that there could be some synergistic effect between the hematopoietic compartment and non-hematopoietic compartment under LRBA-deficient condition. We also confirm for the first time that LRBA is required for surface expression of CTLA4 by Treg cells in the lamina propria of the LI, which has not been assessed in LRBA-deficient humans and was not observed in the *oscar* mutants of LRBA deficiency identified as part of an ENU mutagenesis screen for colitis susceptibility loci (13). We also show for the first time that LRBA plays a crucial role in promoting the production of the key cytokines, IL22 and IL17, produced by ILC3s, which are essential to maintain the gut epithelial barrier and homeostasis in the LI. Finally, we show that SHIP1 agonism can at least partially mitigate DSS-induced colitis in the LRBA-deficient hosts.

These findings reveal that LRBA is not required for the development, maturation, or intestinal trafficking of Treg cells or ILC3 but rather is required for their secretion of key immunoregulatory and effector molecules required for gut homeostasis. LRBA performs this function by promoting either the surface expression of a key immunoregulatory receptor (CTLA4) or the production of key cytokines known to support and mediate repair of the gut epithelium (IL22) or that promote responses to pathogenic microorganisms in the gut (IL17). Thus, LRBA’s role in promoting and coordinating immune cell expression or secretion of key effector molecules (2, 4) can now be extended to regulate innate immune cell types like ILC3. Recently, an analysis of another *Lrba^−/−^* mouse strain has revealed that LRBA has a similarly precise role in promoting surface deposition of the heterotrimeric G-protein Golf in olfactory neurons with impaired olfaction resulting from LRBA deficiency, indicating that LRBA has a comparable regulatory role in the nervous system (42).

Our findings and those of Lo et al. (18) and Wang et al. (13) suggest that the IBD, which occurs in LRBA-deficient patients and mice, is multifactorial and thus more complex than simply loss of CTLA4 expression by Treg cells or hyper-responsiveness to TLR ligands by DC present in the gut milieu. Although we provide evidence here that CTLA4 expression is diminished in gut Treg cells, we also find that the expression of IL17 and IL22 by gut resident ILC3 in response to DSS challenge is compromised. Both IL22 and IL17, and the ILC3 that produce them, play a critical role in the maintenance of homeostasis in the gut (32–35). IL22 and IL17 regulate many aspects of intestinal barrier function including the production of anti-microbial peptides, mucus production, permeability, and cell proliferation, which are crucial to maintain barrier integrity and provide protection against microbial invasion (43, 44). However, dysregulated production of these two cytokines is also associated with inflammation and cancer (43, 44). Our findings that SHIP1 agonism can mitigate lethal colitis in LRBA-deficient hosts are consistent with the increased activation of the PI3K/Akt axis in DC that was demonstrated in the *oscar* mutants (13). SHIP1 has been shown to have a crucial regulatory in most or all hematolymphoid cell lineages (14, 45). As SHIP1 agonism did not prevent the LRBA KO mice from succumbing, this could be due to the inability of SHiP1 agonism to mitigate the loss of CTLA4 function in T cells or diminished ILC3 function (e.g., IL17 and IL22 production) or possibly some other immune dysfunction that contributes to lethal disease in the context of a chemically compromised gut epithelial barrier. Alternatively, the potency and/or bioavailability of the AQX-MN100 agonist may not have been optimal for the full protection of LRBA KO hosts. Further study of SHIP1 agonists in non-lethal models of LRBA colitis, as utilized in the *oscar* mutants, is however worth investigating. These findings also provide insights into the basis of IBD in LRBA-deficient patients and suggest possible molecular-based treatments for this disease (e.g., SHIP1 agonism). Thus, although our studies shed further light on possible causes of IBD in LRBA-deficient patients and suggest it is a disease with multiple causes, there is still the possibility that in humans SHIP1 agonists might be able to mitigate the disease. Perhaps they could also be combined with therapies like abatacept or sirolimus to increase survival and reduce disease burden, perhaps as a prelude to preparing patients for transplant from a suitable HLA-matched BM donor since disease burden prior to transplant influences the eventual outcome of BMT procedures in LRBA-deficient patients (22).

## Data Availability Statement

The original contributions presented in the study are included in the article/**Supplementary Material**. Further inquiries can be directed to the corresponding author.

## Ethics Statement

The animal study was reviewed and approved by IACUC, SUNY Upstate.

## Author Contributions

WK, RS, SF, and CP conceived and designed the study. RS, SF, CP, NS, and RE acquired, analyzed, and interpreted data. SM and JC prepared AQX-MN100. WK, RWE, RS, SF. RS, SF drafted the manuscript; WK, RS, SF, CP, SM, JC, and RE critically revised the manuscript for important intellectual content. RS and SF performed statistical analyses. WK obtained funding and supervised the study. All authors listed have made a substantial, direct, and intellectual contribution to the work and approved it for publication.

## Funding

This work was supported in part by NIH grant RO1 AG059717 to WK.

## Conflict of Interest

The authors declare that the research was conducted in the absence of any commercial or financial relationships that could be construed as a potential conflict of interest.

## Publisher’s Note

All claims expressed in this article are solely those of the authors and do not necessarily represent those of their affiliated organizations, or those of the publisher, the editors and the reviewers. Any product that may be evaluated in this article, or claim that may be made by its manufacturer, is not guaranteed or endorsed by the publisher.
