# Supplementary material for: LRBA Deficiency Can Lead to Lethal Colitis That Is Diminished by SHIP1 Agonism
Source: Front Immunol. 2022 May 4;13:830961. doi: 10.3389/fimmu.2022.830961 (PMC9116273; doi:10.3389/fimmu.2022.830961)
Supplement: Supplementary file 1 [file DataSheet_1.pdf]

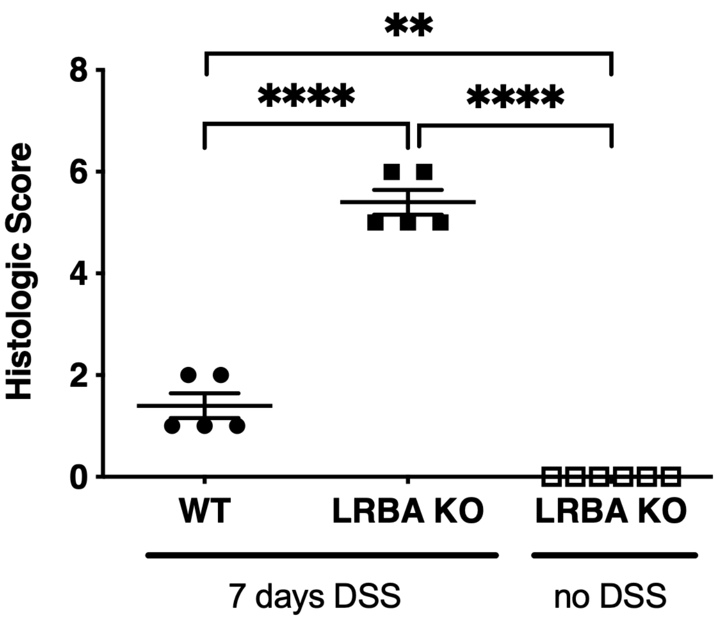

Supplementary Figure 1. LRBA KO mice does not show any histological pathology of colitis in LI under homeostatic conditions without DSS challenge. Figure shows **h**istologic scoring of colitis in naïve LRBA KO mice not given DSS in drinking water compared to WT and LRBA KO mice given DSS in drinking water for 7 days as shown in main figure 1**.** **P<0.01, ****p<0.0001

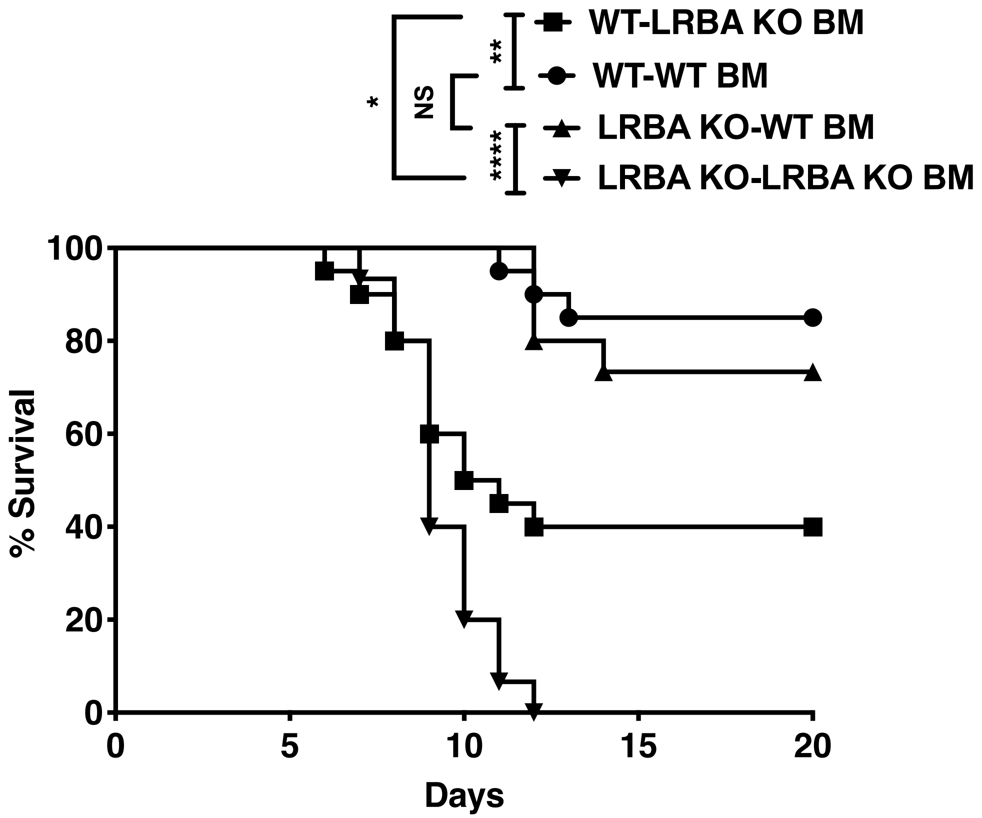

Supplementary Figure 2. Susceptibility to colitis in LRBA KO mice is associated with hematopoietic compartment. Lethally irradiated WT mice were reconstituted with WT(WT-WT BM) or LRBA-/- bone marrow cells (WT-LRBA KO BM) or Lethally irradiated LRBA KO mice were reconstituted with WT(LRBA KO-WT BM) or LRBA KO BM (LRBA KO-LRBA KO BM) (as shown in main Fig 2). After 8 weeks mice were given 3% DSS in drinking water for 7 days and were monitored daily for survival. *p<0.05, **P<0.01, ****p<0.0001

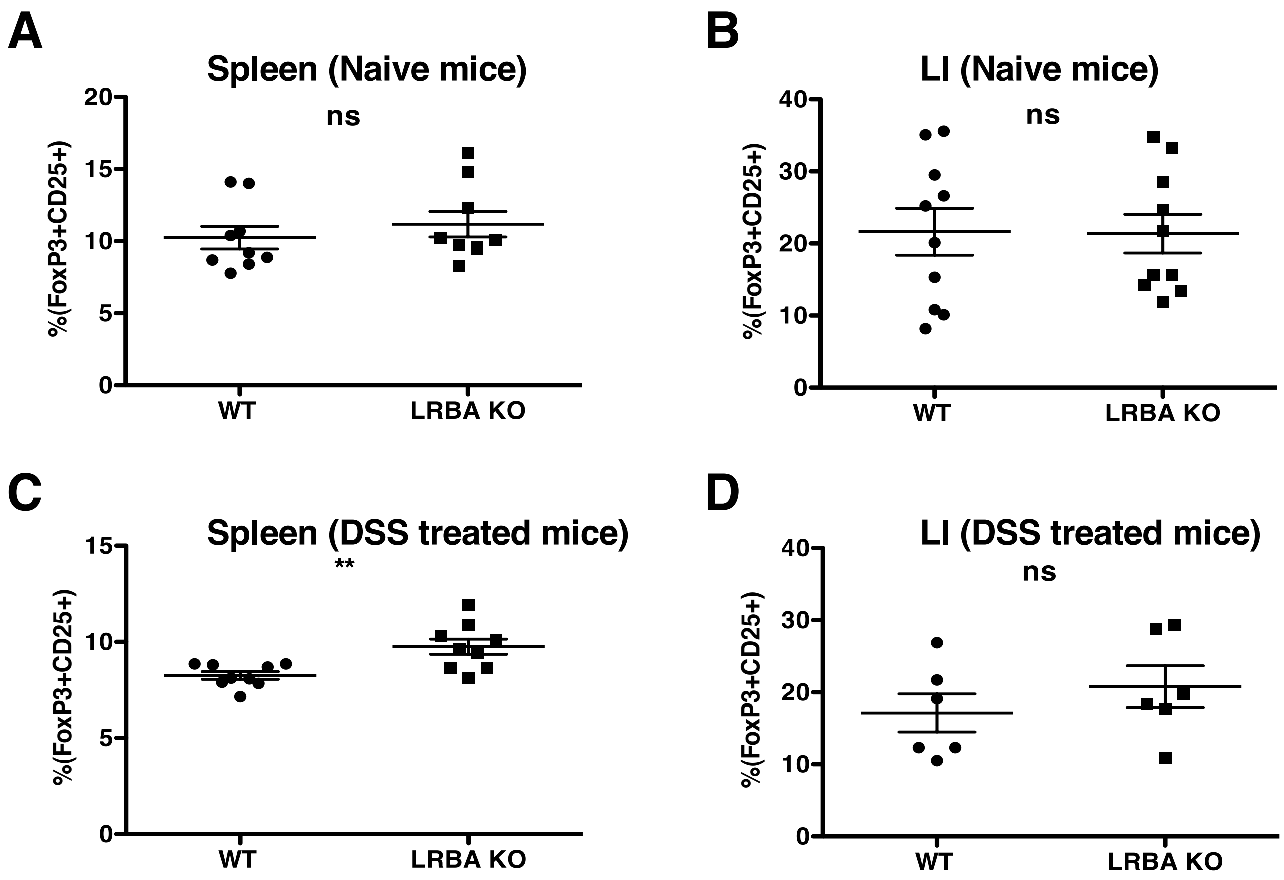

Supplementary Figure 3. Treg cell frequency in naïve and DSS treated LRBA KO and WT mice. Scatter plot showing percentage of FoxP3+CD25+ Tregs from spleens**(A)** and LI **(B)** of naïve untreated WT and LRBA KO mice. **C-D** WT and LRBA KO mice were give 3% DSS in drinking water for 5days and were analyzed for Tregs on 6th day. Scatter plot show percentage of Foxp3+CD25+ Tregs from spleens **(C)** and LIs **(D)** of DSS treated mice. **A-D** Foxp3+CD25+Tregs were gated on live CD3+CD4+ cells. **A-C** pooled data from three experiments is shown with n=9-10; **D** pooled data from two experiments is shown n=6. **ns**(non-significant p value, p>0.05), **p<0.01.

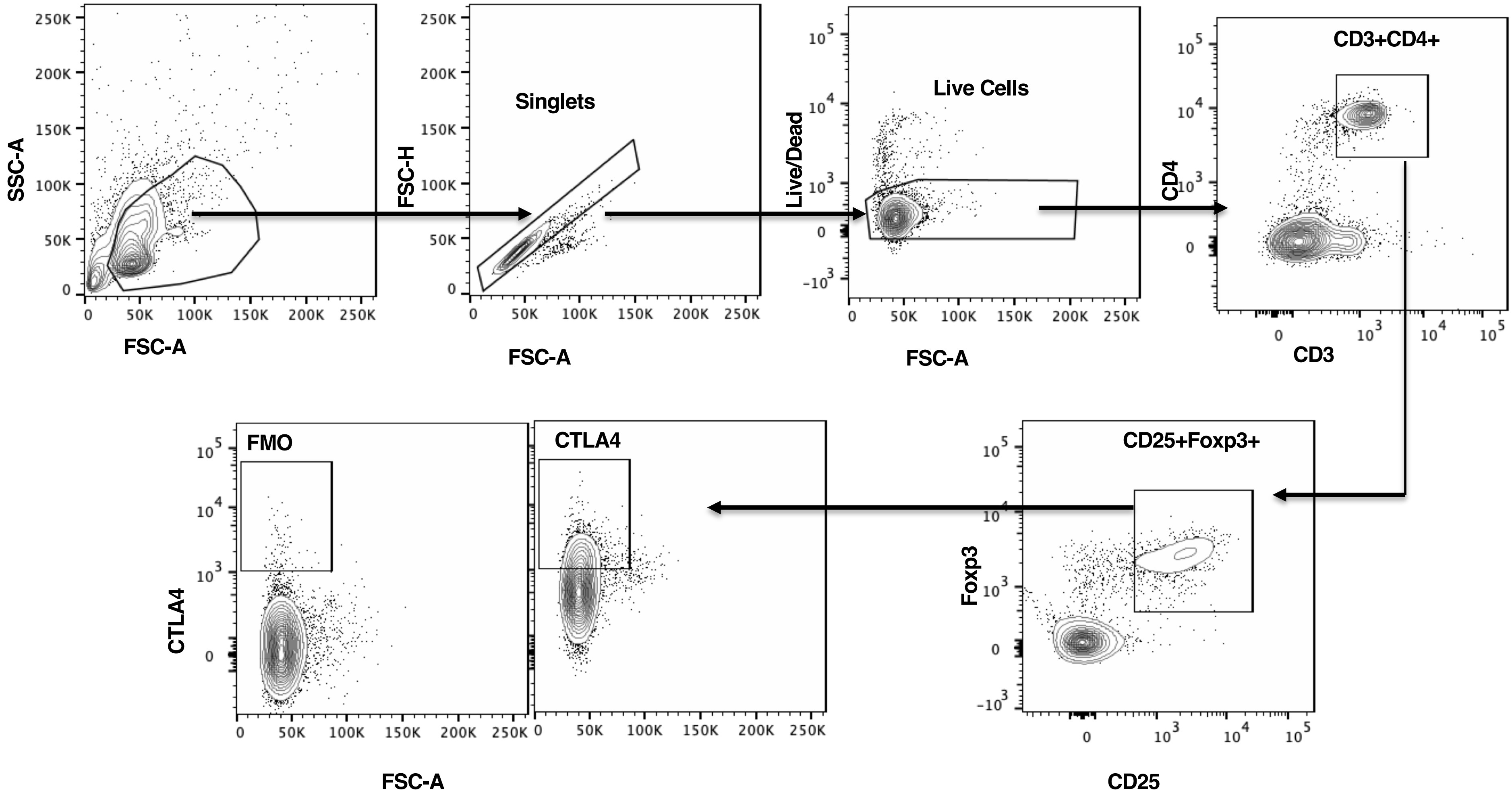

Supplementary Figure 4. Gating strategy to check CTLA4 expression. Flow cytometry plots showing gating scheme used to access CTLA4 expression on Tregs by IC flow from spleen. As shown FMO control was used to set the gate.

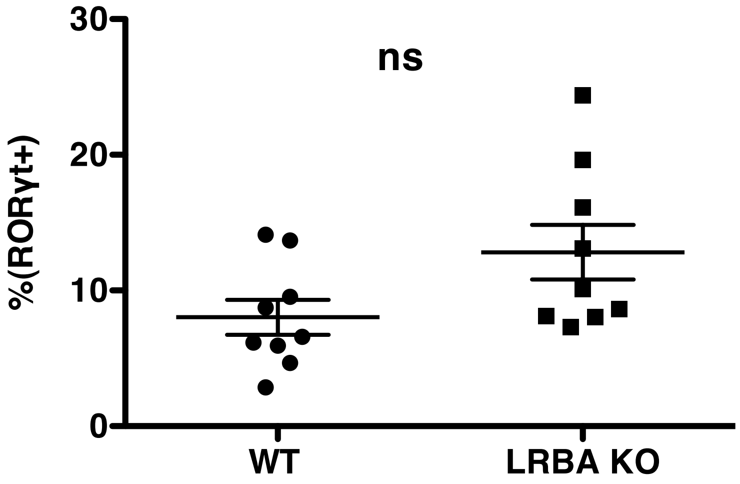

Supplementary Figure 5. Frequency of large intestine lamina propria RORγt⁺ ILC3s is unaffected by LRBA-deficiency. Scatter plot showing frequency of large intestine lamina propria RORγt⁺ ILC3s from 5 day DSS treated mice. Large intestine lamina propria cells were stimulated with PMA/ionomycin for 4 hr and intracellular staining for RORγt was performed. Cells were gated on live, Lin-CD45+Thy1.2+IL7Rα+ cells. Pooled data from three experiments is shown n=9. **ns** (non-significant p value($p>0.05$) n=9 .
